# Neurorobotic reinforcement learning for domains with parametrical uncertainty

**DOI:** 10.3389/fnbot.2023.1239581

**Published:** 2023-10-25

**Authors:** Lana Amaya, Axel von Arnim

**Affiliations:** Department of Neuromorphic Computing, Fortiss-Research Institute, Munich, Bavaria, Germany

**Keywords:** domain randomization, neuromorphic computing, neurorobotics, reinforcement learning, robot control, spiking neural networks

## Abstract

Neuromorphic hardware paired with brain-inspired learning strategies have enormous potential for robot control. Explicitly, these advantages include low energy consumption, low latency, and adaptability. Therefore, developing and improving learning strategies, algorithms, and neuromorphic hardware integration in simulation is a key to moving the state-of-the-art forward. In this study, we used the neurorobotics platform (NRP) simulation framework to implement spiking reinforcement learning control for a robotic arm. We implemented a force-torque feedback-based classic object insertion task (“peg-in-hole”) and controlled the robot for the first time with neuromorphic hardware in the loop. We therefore provide a solution for training the system in uncertain environmental domains by using randomized simulation parameters. This leads to policies that are robust to real-world parameter variations in the target domain, filling the sim-to-real gap.To the best of our knowledge, it is the first neuromorphic implementation of the peg-in-hole task in simulation with the neuromorphic Loihi chip in the loop, and with scripted accelerated interactive training in the Neurorobotics Platform, including randomized domains.

## 1. Introduction

Neuromorphic hardware is characterized by high energy efficiency, low latency, and adaptability. These characteristics are of critical importance in robot control tasks, where real-time decision-making is a key factor, where energy is a constraint, and where environments are dynamic.

This hardware can efficiently run brain-inspired models known as spiking neural networks (SNN) (Hodgkin and Huxley, 1952; Arthur and Boahen, 2011). However, optimally training these networks remains an open question, partly on account of a lack of understanding for how the human brain learns (Lillicrap et al., 2020).

Different approaches have been proposed in the last years to cope with this challenge. Studies such as Bellec et al. (2020) and Yang et al. (2023) have investigated biologically plausible learning approaches. The same authors provide new perspectives for efficiently learning in SNNs by using (a) the minimum error entropy criterion (Yang et al., 2022) and (b) the information theoretic learning approach (Yang and Chen, 2023). In any case, how the brain manages to efficiently learn with its local constraints remains a conundrum.

Furthermore, training neural networks becomes specially challenging in the case of non-static or interactive learning, wherein the dataset distribution depends on the model being optimized itself. This is true of complex robot control tasks, where reinforcement learning (RL) is an usual approach for model fitting. When those models are SNNs, the formalism is known as spiking reinforcement learning. Recent such as Tang et al. (2020, 2021) have investigated this formalism for learning in environments with continuous state and action spaces, as usually seen in robotics.

Robot learning can be complicated by the additional challenges of limited hardware resources, time and cost constraints, both leading to too few learning data. Therefore, simulators are a key element for collecting non-static data. However, simulations may not model reality with sufficient accuracy, and the learnt behaviors may not generalize to the real domain. With additional effort though, this problem, known as the sim2real gap, can be dealt with. System identification, domain adaptation, and domain randomization are techniques that have been proposed in literature (Inoue et al., 2017; Weng, 2019; Beltran-Hernandez et al., 2020; Kaspar et al., 2020) to reduce this gap.

To approach these issues, a number of integration frameworks have been developed. The most relevant may be the neurorobotics platform (NRP), developed as part of the Human Brain Project. The NRP is an open source integration framework for robot and SNN simulators, intended for in-silico neurorobotic experimentation (Falotico et al., 2017). Herein, we extend its functionalities to integrate neuromorphic hardware (Loihi) in the loop to use gradient information from multiple simulations in learning algorithms and to randomize environments to learn in situations characterized by domain uncertainty or variability. Using the Loihi chip in the simulation loop of the NRP has been previously implemented (without learning) by Angelidis et al. (2021); however, this was using the Nengo framework which adds considerable overhead and architectural constraints. In this study, the network architecture is customizable, the synapric weights are learned, and we use a direct integration with Loihi's low-level SDK NxSDK over multiple simulation episodes.

In this study, as a proof of concept, we also solve a model-free robotic force-guided object insertion task. Object insertion has most of the elements of a traditional robotic task, and therewith, our approach can be easily generalized to a wide array of robotic use cases. The programming of robot systems to handle object insertion tasks in modern manufacturing scenarios (e.g., plugging in connectors in electronic devices for the automotive industry or clipping elements in industrial assemblies) is challenging due to complex contact dynamics and friction.

There are still many knowledge gaps around spiking RL for robotics. Integrating neuromorphic hardware with robot simulation environments and reinforcement learning algorithms is a key step to facilitate research in this emerging and promising field of study. Doing this can contribute in the near future to solve challenging real robotic tasks in innumerate robotic fields from industrial to assistive robotics.

## 2. Materials and methods

In Subsections 2.1 and 2.2, we describe the simulation and integration environment as well as the neuromorphic hardware. Then, the methods are described, including the spiking neural networks used, the reinforcement learning algorithm, the robot control and simulation setup, as well as the connectivity between these and the setup for training neurorobots in domains with parametric uncertainty.

### 2.1. Simulation tools and frameworks

The neurorobotics platform v3 (NRP)^1^ is used in this study as an integration environment for running simulations and defining learning schemes. The NRP has been developed as a major deliverable of the Human Brain Project (Falotico et al., 2017). It allows to connect customized SNNs (brain models) to robot environments. Additionally, experiments can be performed on a cluster of high-performance computers. These simulations can be run on a local or remote machine; in both cases, users can create experiments, simulate them, visualize them, and interact with them through an intuitive graphical users interface.

The NRP consists of six main components:

- the brain simulator, which runs a spiking neural network over different possible backends, including hardware,
- the world simulator (based on Gazebo^2^),
- the brain interface and body integrator (BIBI) for building a communication channel between the former two,
- the closed loop engine (CLE) for orchestrating them,
- a backend exposing a web server with a RESTful API,
- a frontend to provide a graphical interface.

**Figure 1:**
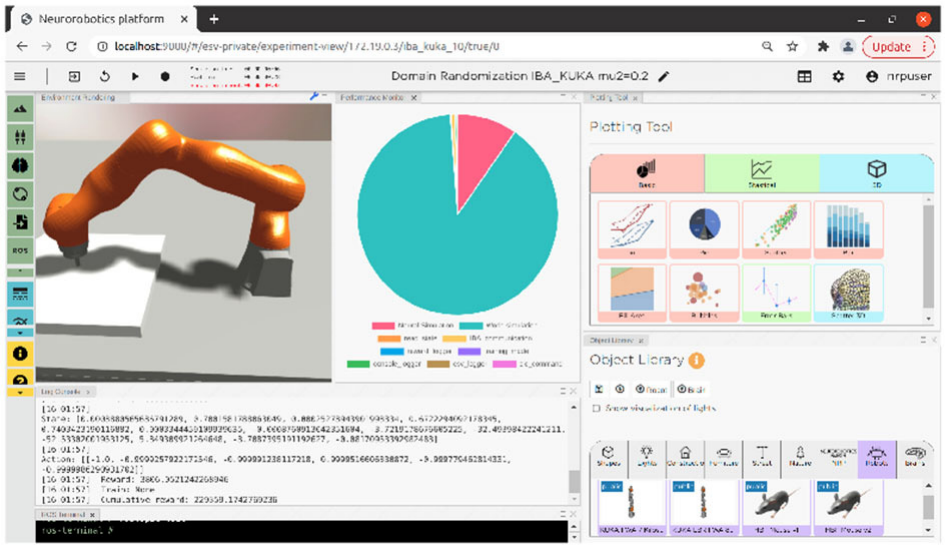
NRP v3 frontend view including environment rendering, performance monitoring, plotting tool, log console, object library, and ROS terminal.

**Figure 2:**
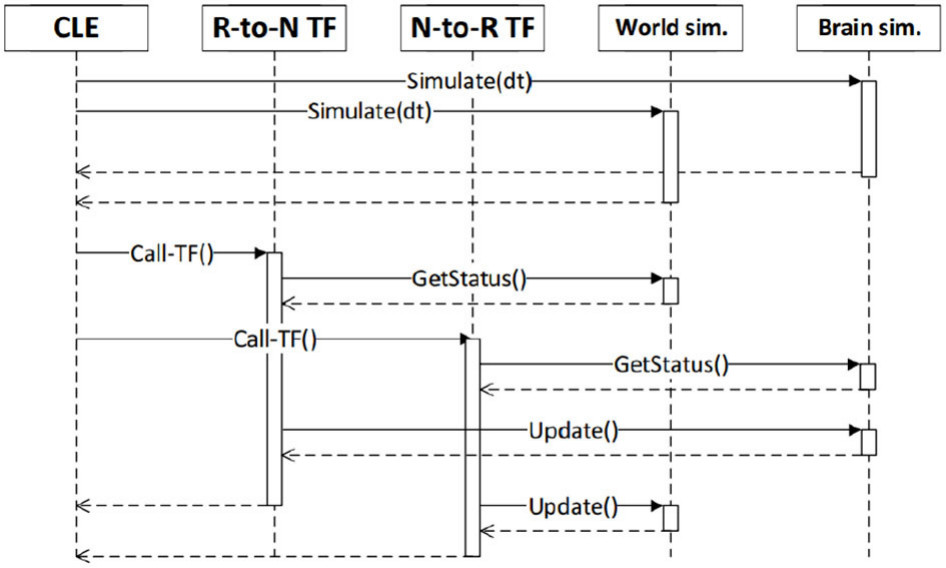
Synchronization between the components of a simulation and user code (robot-to-neuron functions, referred as “R-to-N TF” and neuron-to-robot functions), as orchestrated by the CLE. Reproduced from Falotico et al. (2017).

Figure 1 shows the frontend view and some of its functionalities, while Figure 2 shows how all the NRP elements are timely orchestrated by the CLE. The NRP adds two optional components for more flexible and advanced integration with external software:

- the Integrated Behavioral Architecture (IBA) to execute external code within the simulation loop
- the Virtual Coach (VC): a python interface to the NRP that enables to script simulations

These two extensions of the NRP will be described later.

#### 2.1.1. Brain simulation: the model

The NRP supports executing brain code from a set of frameworks including Nest, PyNN, and Nengo. These supported frameworks are run in the simulation loop and can exchange information with the other components at every execution step. For this study, the SNNs used were defined in PyTorch and NxSDK.^3^ Since these frameworks are supported, we made use of the IBA mechanism to run them as the external code in the NRP.

#### 2.1.2. IBA: embedding external code

The integrated behavioral architecture (IBA) is a mechanism for the execution of external code, including AI frameworks such as PyTorch. With it, unsupported libraries can be embedded in the NRP. For example, it can run Tensorflow code synchronized with the world simulation. The IBA is implemented as a ROS launch file that loads GazeboRosPackages modules. These modules are called by the Closed-Loop Engine (CLE) within each loop iteration of a simulation. We used this mechanism to embed our reinforcement learning algorithm, in both versions, PyTorch on CPU and NxSDK on Loihi 1.

#### 2.1.3. World simulation: the environment

The environment is defined as a Gazebo model simulated in the NRP-backend. Interactions between the world simulation and the brain can be defined via “transfer functions” in the NRP or, in our case, via ROS communication over topics for the IBA brain code. The world environment modeled for this manuscript is described in detail in Section 2.3.

#### 2.1.4. Virtual coach: scripted interactions between simulations

The Virtual Coach is a tool for scripting front-end interactions in the NRP, which allows for defining and running simulations automatically, as well as interacting with them. This python API enables to edit experiments, clone them, launch and control simulation runs, and retrieve data. In this study, we use those capabilities to script closed-learning loops within the NRP.

### 2.2. Neuromorphic hardware

The neuromorphic processor used for running the SNNs in this study is the first generation Loihi research chip (Davies et al., 2018). Loihi is a digital discrete-time neuromorphic chip with a specialized scalable architecture optimized to run and train programmable SNNs. Each Loihi chip contains over 131 thousand neurons partitioned over 128 cores connected in a mesh; every core contains 1,024 neurons, with the cores responsible for allocating synaptic states (128 kB) and managing routing tables (20 kB). The neurons are current-based-synapse leaky-integrate-and-fire neurons (CUBA-LIF). The weights are stored in the neuromorphic core in a compressed format to enable efficient allocation. Additionally, the synapse weights can have variable precision (from one to nine bits).

Neurons communicate to each other between cores based on the routing tables and via spikes, which are 32 bit messages that contain destination addresses, source addresses, and payloads. Both the mesh and the cores are built with asynchronous circuits and communicate event-driven data; even though Loihi has no clock, periodic wave fronts are used for inter-core spike synchronization. Each Loihi chip also has three microcontroller-class x86 processors which can be used for different proposes such as data format conversion and bridging encodings.

More specifically, the neuromorphic hardware used in this study was a Kapoho Bay device with two Loihi chips. It has 256 neuromorphic cores with 262,144 neurons, and 260,000,000 synapses.

### 2.3. The experiment—robotic force-torque-based object insertion

The robotic peg-in-hole task has been established as a benchmark for interactive control strategies in academic and industrial research. This task consists of two phases: search and insertion. The search phase consists in finding the hole position and reaching an initial pose^4^ with the manipulator. The insertion phase involves not only motion but also force interaction with the environment and can be approached through either model-based or model-free strategies. In the case of contact model-based strategies, a contact model analysis decomposes the peg-in-hole task into contact-state recognition and compliant control. On the other hand, contact model-free strategies treat both components as a whole (Xu et al., 2019). In this study, we approach the searching phase from a model-free perspective.

#### 2.3.1. World setup

**Figure 3:**
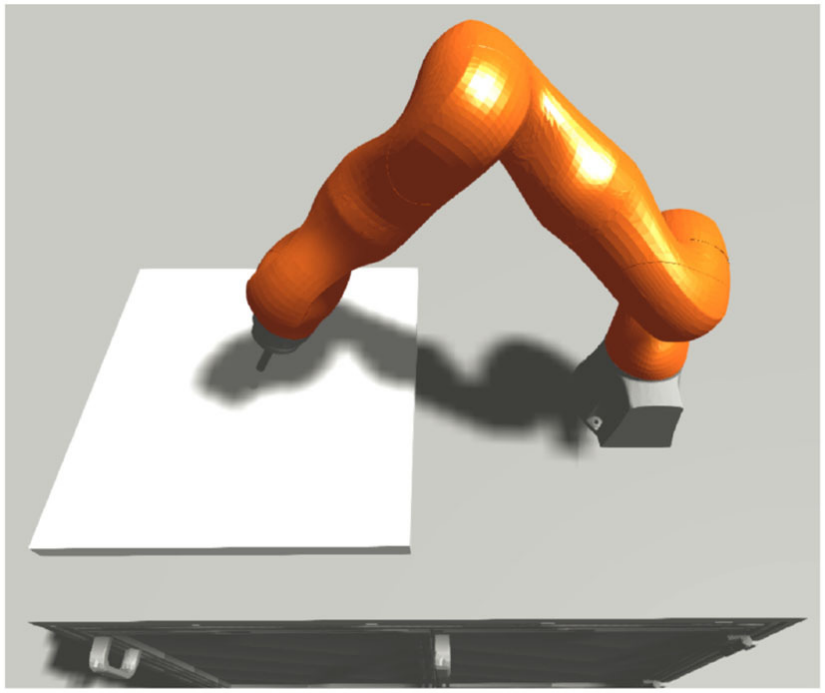
Simulated world setup.

**Figure 4:**
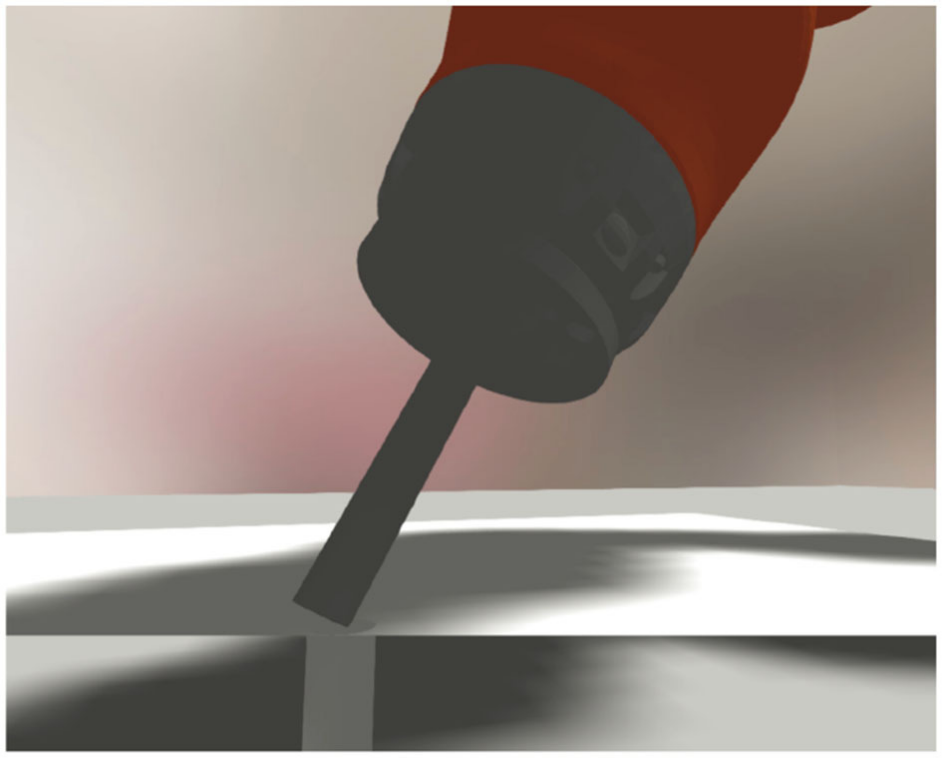
Zoomed-in picture of a sectional cut of the insertion-hole, the peg, the force-torque sensor, and the last joint of the robot.

The world setup for the peg-in-hole task is depicted in Figure 3. We used a round peg with a flat end and a round hole with a 0.5 mm clearance depicted in Figure 4. The platform with the hole is attached to a horizontal plane inside the dexterous space of the robot, and the peg is connected to a force-torque sensor attached to the robot as an end-effector. Both the robot and the insertion platform are located on a cabinet with the robot controller.

#### 2.3.2. Robot and control

The robot used in simulation is a Kuka LBR iiwa 7 R800, a redundant lightweight manipulator equipped with torque sensors at each of its seven joints. A simulated Cartesian Impedance Controller (CIC) was implemented to soften contact interactions in the task space. The CIC controller leads the closed-loop response of the system to simulate the desired spring-damper dynamics in the Cartesian coordinates with respect to the target position and velocities. The control law is given by Equation 1, which can be simplified by setting the desired apparent inertia matrix to match the real Cartesian inertia matrix as in Equation 3.


(1) $$\begin{array}{l} \tau =\;M(q)J_{a}^{-1}(q)\{{\ddot{x}}_{d}-{\dot{J}}_{a}(q)\dot{q}+M_{m}^{-1}[D_{m}({\dot{x}}_{d}-\dot{x})\\ \;+K_{m}(x_{d}-x)]\}\\ \;+C(q,\dot{q})\dot{q}+g(q)\\ \;+J_{a}^{T}(q)\left[M_{x}(q)M_{m}^{-1}-I\right]F_{a} \end{array}$$

(2) $$\begin{array}{l} \tau =\;M(q)J_{a}^{-1}(q)\{{\ddot{x}}_{d}-J_{a}(q)\dot{q}\}\\ \;+C(q,\dot{q})\dot{q}+g(q)\\ \;+J_{a}^{T}(q)\left[D_{m}\left({\dot{x}}_{d}-\dot{x}\right)+K_{m}\left(x_{d}-x\right)\right], \end{array}$$

where τ is the torque command for the robot, *M* is the robot inertia matrix, *M*_*m*_ the desired apparent inertia matrix, *M*_*x*_ the real Cartesian inertia matrix, *C* the Coriolis matrix, *J*_*a*_ denotes the robot analytic Jacobian, *D*_*m*_ the desired damping, *K*_*m*_ is the desired stiffness, *q* is the joint positions, *x* is the current Cartesian pose, *x*_*d*_ is the desired Cartesian pose, and the dot notation denotes the first and second time derivatives.

The Cartesian impedance framework is introduced in Hogan (1985). Albu-Schaffer and Hirzinger (2002) provides an extension of the CIC to lightweight robots with flexible links such as the Kuka iiwa LBR, while Albu-Schaffer et al. (2003) provides an extension of the CIC to redundant manipulators with null space considerations. Additionally, the latter presents two different strategies for tuning the CIC. The second tuning scheme presented therein has been used to tune the controller in this study.

#### 2.3.3. Sim-to-real gap

Simulation models can significantly differ in their behavior with respect to their corresponding real systems. Models of reality used for simulations can diverge from real data. Some of the most critical variables leading to these differences in the field of robotics include object position, dimensions and masses, robot link masses and inertia, surface friction coefficients, controller gains, and damping factors (Weng, 2019). Alternatively, disturbances and sensor noise represent a further source of discrepancies between modeled and real environments.

### 2.4. Implementation of the learning scheme

In order to teach the arm to insert the peg, we make use of RL. We first outlined the Markov decision process (MDP) formalization as well as the associated reward function. The chosen RL approach is then described. With these, we then used the Virtual Coach (Section 2.1.4), together with the IBA (Section 2.1.2), to define complex learning environments requiring software and hardware not supported natively in the NRP.

#### 2.4.1. Utilized Markov decision process

The MDP can be defined as a tuple in the form $\langle S,A,R,P,\rho_{0}\rangle$, where S is the state space, A is the action space, R:S×A×S→ℝ is the reward function, P:S×A→ℙ(S) is the transition probability function, and ρ_0_ is the initial state distribution.

For the insertion task, the state space S is a 13-dimensional set of continuous variables including the robot's current coordinates, alongside the measured forces and torques. The action space A is a 6-dimensional set of continuous variables, namely, the target values for the Cartesian impedance controller described in the former sections. The reward function R is defined as


(3) $$R=w_{1}||f_{d}-f|{|}_{2}+w_{2}||\tau_{d}-\tau |{|}_{2}+w_{3}||z_{d}-z|{|}_{2}$$

Here *f*_*d*_, τ_*d*_, and *z*_*d*_ are the desired force, torque, and depth. Similarly *f*, τ, and *z* are the measured values for these three quantities. The weights *w* allow us to adjust the importance we assign to each of these terms. Recall that the goal is to learn insertion based on the force-torque profile measured by a sensor attached to the end-effector (the peg). This is the motivation behind the first two terms. The third term serves to quickly teach the arm to remain on the table. Without this term, the number of training episodes becomes impractically large.

The transition probability function P is given by the world dynamics of the randomized domain set, and the initial state distribution ρ_0_ is defined by a normal distribution centered on the approach pose.

#### 2.4.2. Reinforcement learning implementation

We now describe the implementation and the detailed learning setup. The reinforcement learning cycle is composed of the agent interacting with the environment. The latter has been described in Section 2.3.1 and is simulated in Gazebo within the NRP. The simulator takes an action *a*, and after one step, it generates the next state *s*. Meanwhile, a policy dictates the actions *a* for the agent based on *s* to maximize a reward. The policy is implemented with the IBA to run spiking neural networks in classical and neuromorphic hardware both in inference and in training modes. For this, we activated a special virtual environment in the IBA start process and run the models (with NxSDK for On-Chip spiking actor network, and pytorch, scikitlearn for the other networks) at every simulation/CLE step.

**Figure 5:**
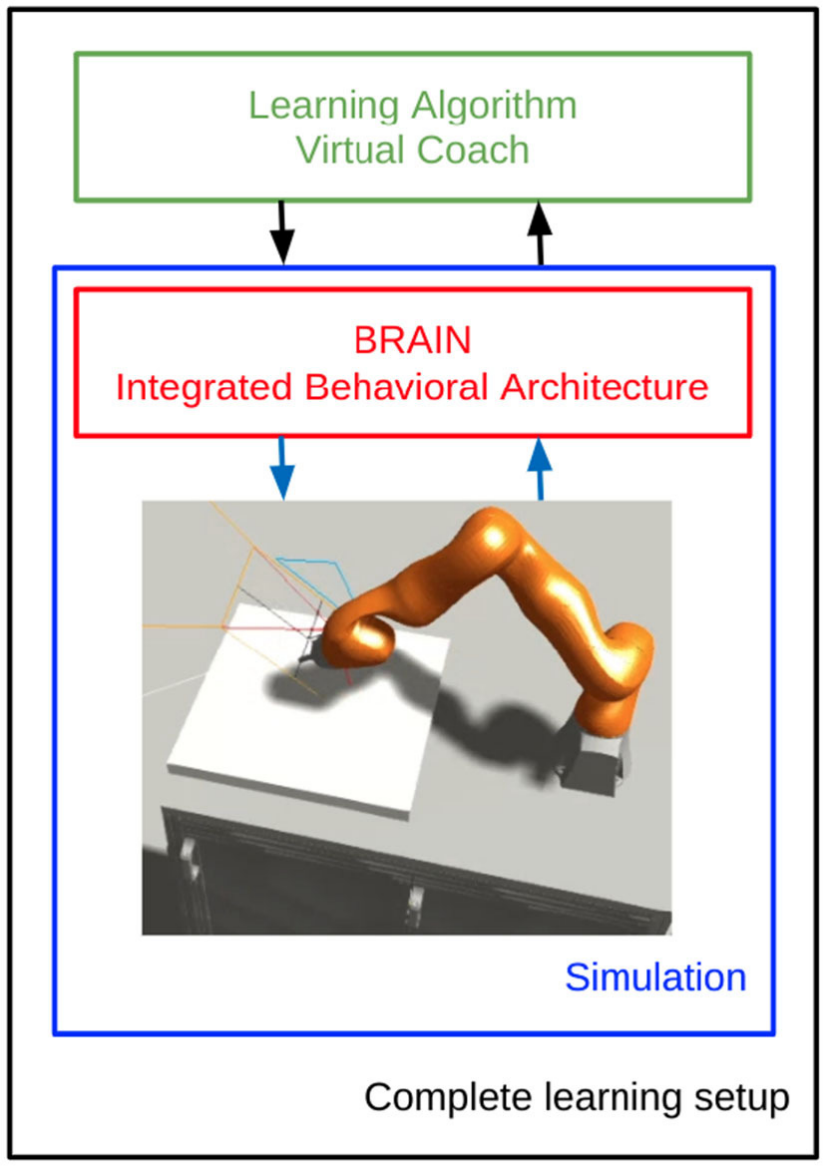
Learning architecture scheme in the NRP. Refer to the pseudo-code in Algorithm 1 and to the schema in Figure 8 for a more detailed description.

This reinforcement learning loop repeats until it completes an episode, then the virtual coach script handles the beginning and end of multiple simulations, gathers the data to feed them to the reinforcement learning algorithm, and updates the models accordingly, as sketched in Figure 5. This includes updating the policy and the value function. The specific update rules, models, and further details are described in the following sections.

#### 2.4.3. Soft actor critic

In this study, the reinforcement learning algorithm used is a version of soft actor critic (SAC) (Haarnoja et al., 2018), which optimizes a stochastic policy in an off-policy fashion. The policy is trained to maximize a trade-off between expected return and entropy. The intuition behind entropy-regularized RL is succeeding at the task while acting as randomly as possible. This contributes to preventing the policy from prematurely converging to an undesired local optimum.


(4) $$\begin{array}{c} \pi^{*}=\;\arg\;\underset{\pi }{\max}\;\underset{\tau ∼\pi }{\text{E}}[\sum_{t=0}^{\infty }\gamma^{t}(R(s_{t},\;a_{t},\;s_{t}+1)\\ \;+\alpha H(\pi \;(\cdot |s_{t})))] \end{array}$$

SAC achieves state-of-the-art performance that surpasses previous on-policy and off-policy methods in sample efficiency and asymptotic performance. Moreover, SAC is more stable and shows more robustness to different random seeds than other off-policy algorithms. Therefore, SAC is a promising candidate that could learn in real robotic applications.

**Figure 6:**
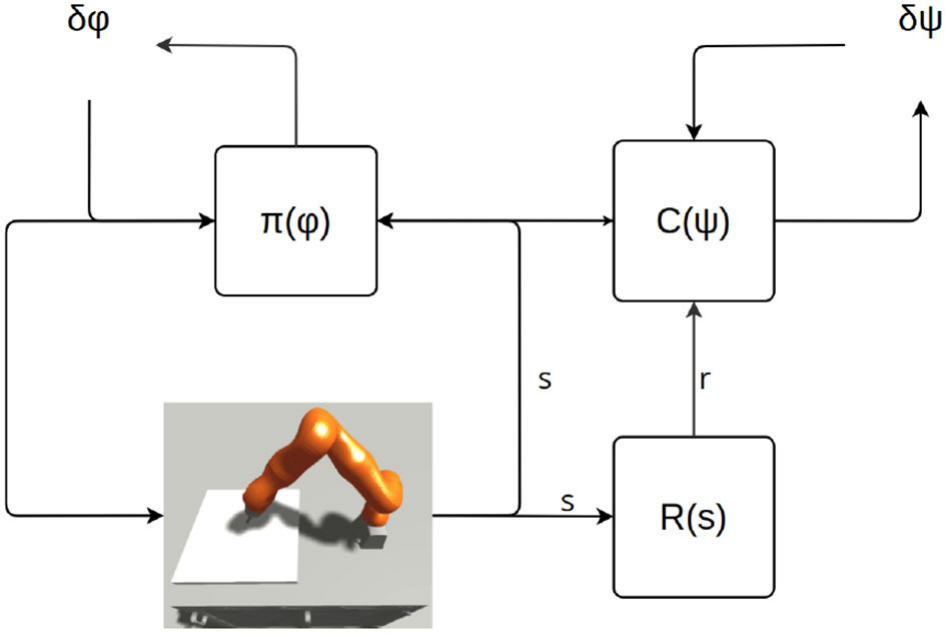
Learning scheme. “R” is the reward function. Π is the policy. Φ represents the parameters of the policy. “C” stands for “critic,” and depending on the actor-critic architecture, it could be a different set of value functions and action-value functions. Ψ represents the parameters of the critic-model(s).

Figure 6 roughly illustrates the structure of an actor Critic RL algorithm.

#### 2.4.4. Spiking implementation—PopSAN

Tang et al. (2020) uses SNNs to implement continuous deep RL. Competitive performance is shown through the use of a spiking version of DDPG (Deep Deterministic Policy Gradient). Note that although the actor network is spiking, the critic is non-spiking since its not used for inference and thus does not have to be ported to neuromorphic hardware. PopSAN (Tang et al., 2021) expands upon the results of RateSAN (Tang et al., 2020) with, among others, a spiking version of SAC incorporating population coded input instead of rate coded input networks.^5^ Our study is based on the PopSAN architecture, which we adapt for the NRP and the robotic insertion task. The networks are architectured as follows. The spiking actor network is a fully connected SNN with LIF neurons and layer sizes 160 × 256 × 256 × 60. The critic networks are fully connected ANNs with ReLU activation functions in the hidden layers and hyperbolic tangent activation functions in the output layer. Both Q-networks have sizes 22 × 256 × 256 × 1. This hybrid training architecture is best implemented on cpu with conventional frameworks. After training, the spiking actor is then ported to the Loihi chip for testing in real neuromorphic hardware for inference, while we do not need the critics anymore.

#### 2.4.5. Domain randomization for robust agents

Domain randomization in the field of robotics is commonly used as a strategy for closing the simulation-to-reality gap (sim2real). It involves multiple training runs, each with randomized parameters to mirror the distribution of the real world domain. In other words, the agent is trained to perform well over a range of situations. Typical dynamic parameters that are commonly randomized include object dimensions and masses, robot link masses and inertia, surface friction coefficients, controller gains, and damping factors (Weng, 2019). Alternatively, disturbances can be added during the training process to reach a similar result. Perturbations could be applied on sensor values, rewards, or even on other parameters that are not generally susceptible to perturbations (i.e., gravity). This can also result in more robust learning.

**Figure 7:**
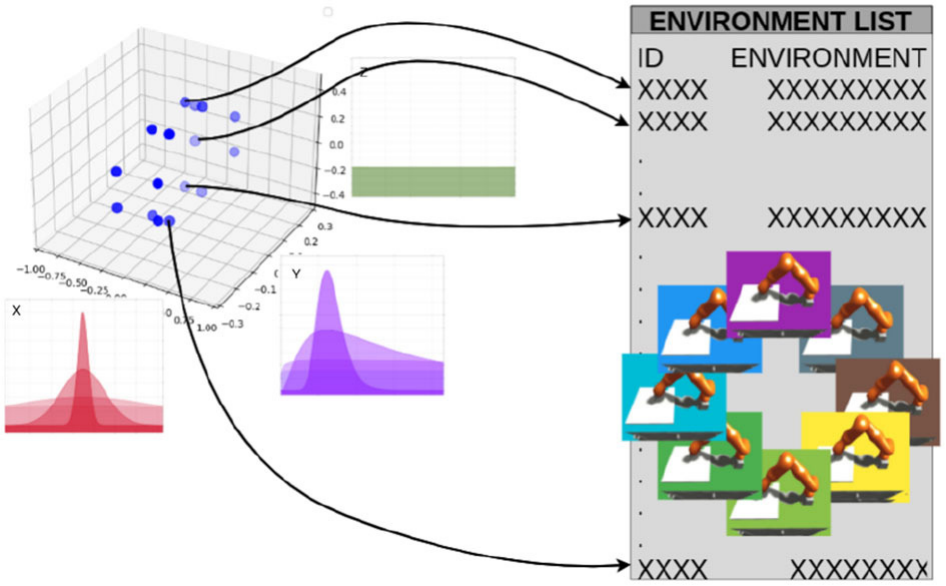
Sampling mechanism: each variable is a parameter that can be randomized. For each dimension of the parameter space, Gaussian and log-Gaussian distributions can be selected as sampling schemes.

This study implements parametric domain randomization in the NRP via the Virtual Coach (Section 2.1.4). The training script requires a list or a multidimensional array (defined in numpy) with the experiment IDs. Figure 7 depicts a list of environments in which all dimensions are already sampled from the distributions and collapsed to one dimension. However, it is also possible to use a multidimensional array as an input, where the elements *ijk* represent the experiment-ID of the environment with the corresponding *ijk* parameters. For instance, the *x, y, z* coordinates could represent the friction coefficient μ = *i*∈*X* = {0.08, 0.36, 0.80}, end-effector mass *m* = *k*∈*Z* = {κ/2|κ∈[1..5]}, and controller gain *k*_*p*_ = *j*∈*Y* = {2υ+1|υ∈[0..4]}.

In the simplest case, a list with experiment IDs could be used instead of an array. This list or multidimensional array could be generated by hand or via scripts. Given the environment list, the training script selects domains based on four sampling schemes: sequential, uniform, normal, and custom defined. In the case of sequential sampling, the order in the list is used to choose the environments. In the uniform case, all source environments have the same likelihood to be chosen, but there is no imposed order. In the normal case, the sampling parameters are chosen from a Gaussian distribution and then approximated to the closest environment with that parameter as there is no continuum of environments (notice that this will result only in an approximation of strict normal sampling). Finally, additional sampling distributions could be defined and passed as functions to the method.

Furthermore, the definition of the environments could be used to generate more complex sampling distributions: for instance, if the parameters are equidistant in a logarithmic space and a Gaussian sampling strategy is selected, then the sampling scheme would actually be based on a log-normal distribution.

More specifically, to test the approach, we trained single models with extreme friction parameters μ_1_ = μ_2_ = 0.08 and μ_1_ = μ_2_ = 0.8. A generalized agent trained for the characterized friction parameters μ_1, 2_ = 0.38 ± 0.4 with domain randomization and we compared the results.

**Figure 8:**
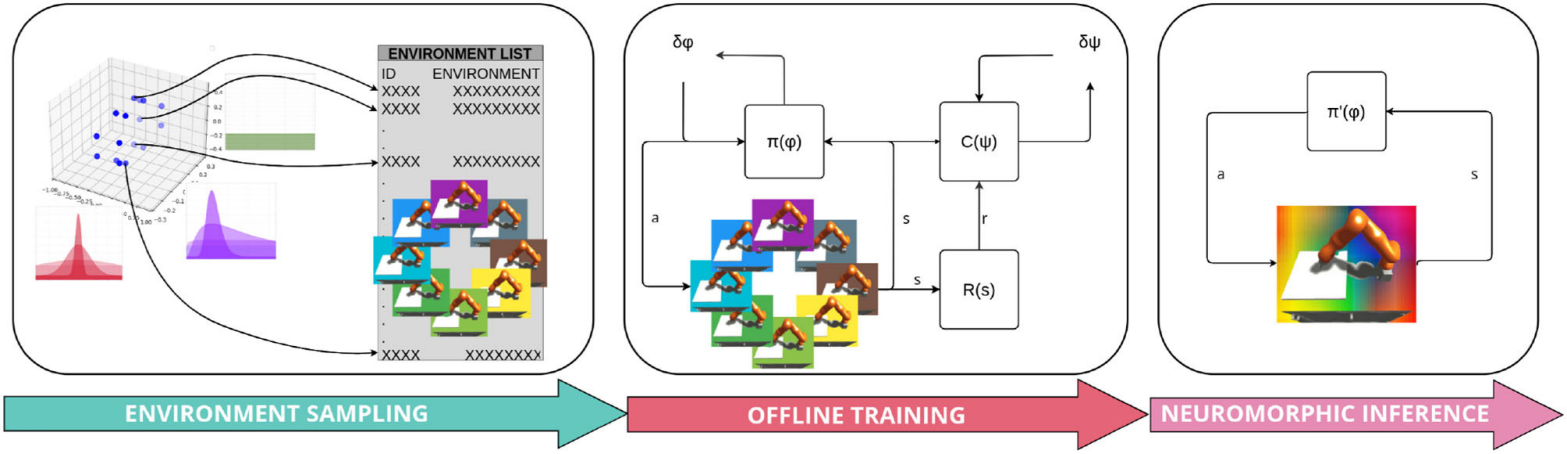
Full overview of the training pipeline.

We also implemented a sampling scheme in which wider distributions with more domain variability are progressively selected based on the epoch-number or epoch-performance. This allows the agent to first learn in an easy environment, and once a successful behavior has been reached, the complexity is incremented by progressively extending the variance over the source domain parameter set. One can imagine this as a sort of “training wheels,” which are removed when an agent becomes more adept, inspired from Heim et al. (2018). However, there may be scenarios which benefit from preemptively consideration of the whole range of domain variability. Figure 8 shows an overview of the whole learning approach.

#### 2.4.6. Injecting prior knowledge

As soft actor critic is an off-policy algorithm, the data used for the training can be collected by an arbitrary sampling distribution. We use this fact to bootstrap training with a memory buffer collected by a teacher-agent and relabel it for the new agent. We use a policy trained on a simpler setup with additional hints and information that are not available to the real agent. The teacher agent is able to visit regions of the state space that a random agent at the beginning would not be able to visit.^6^ Even though the policies differ, pre-training with this data provides at least a notion of the values related to the states. This would be equivalent to transferring the value functions if the state spaces of both agents were to overlap completely.

#### 2.4.7. Consideration of neuromorphic hardware constraints

Once the architecture of the SNN and the synaptic weights have been decided on, implementing the SNN can be easily done in neuromorphic hardware. More specifically, for porting to Loihi 1, we implement the network architecture with NxSDK and load the equivalent synaptic values from the PyTorch models, as done previously in Tang et al. (2021).

One important consideration is the discrepancy in the trained model and the one implemented in hardware. Different hardware alternatives present different resource limitations and discrepancies between their ideal SNN models and simulated versions. For instance, Loihi, being a digital chip, uses neuron states and synaptic weights which accuracy is typically limited to 8 bits, while the LIF model is continuous and its PyTorch simulated version has full precision weights (32 bits).

To deal with this problem, Akl et al. (2021) implements a spiking version of DQN where the weights are kept quantized in the forward pass but treated as real values during the backward pass. This is achieved by using a technique known as the straight-through estimator. Even though this method can only be applied in environments with discrete action spaces and does not consider the neuron state quantization, its results show considerable on-chip performance improvements. Additionally, the concept can be extended to other RL algorithms without major changes.

Additional hardware constraints include the maximum amount of networks per neural core, max amount of neural cores, as well as the max fan-in and max fan-out for core-to-core connections. These values are described in detail in Davies et al. (2018, 2021). Similarly, other alternative neuromorphic chips present different constraints. It is important to consider these to ensure reliable results when porting to hardware. In essence, the process consists in making the model resemble the targeted hardware and consider all its limitations before training or at least approximate the model and assert that the discrepancies do not generate a considerable difference in performance.

## 3. Results

The materials and methodology presented previously have been used to train robust agents offline and port them to neuromorphic hardware, leading to the following results.

### 3.1. Spiking neural network—generic training

**Figure 9:**
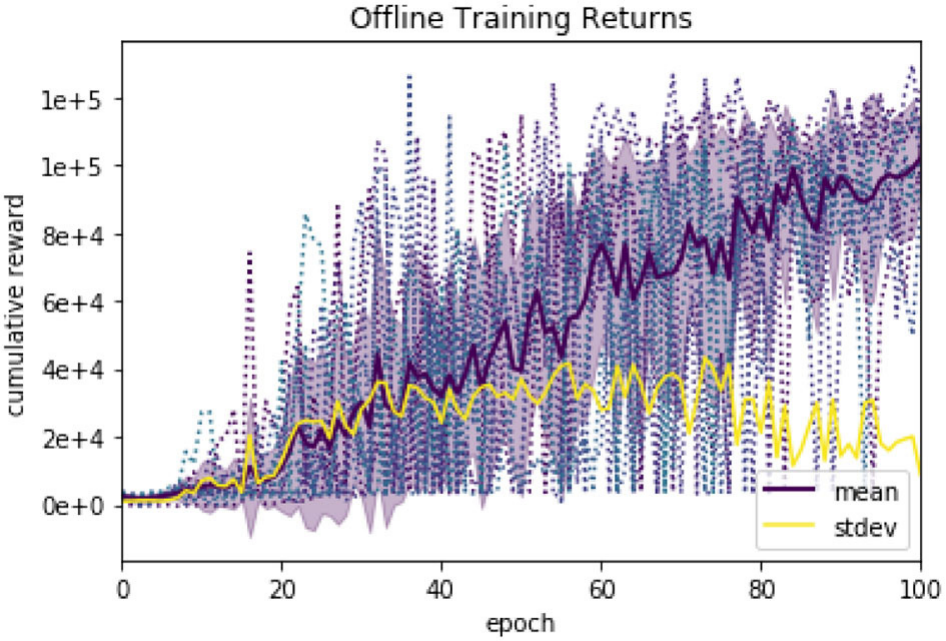
Learning curves show the results of training with 20 random seeds, the mean performance and the standard deviation.

The training was run for 100 epochs, with 500 episodes and 2000 interactions each. Figure 9 shows the results with training 20 agents with different random seeds in terms of mean return and corresponding standard deviation. We found a 100% insertion success rate across 50 runs using a trained policy. A video of the trained agent can be found in the Supplementary material Video 1.

Figure 9 shows the training profile of 20 different random seeds.

### 3.2. Inference on neuromorphic hardware

**Figure 10:**
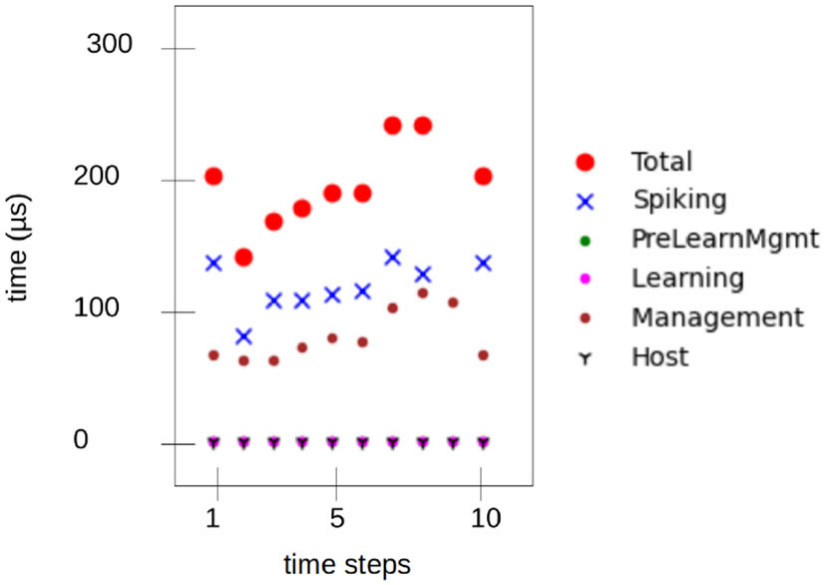
SNN execution time on Loihi 1 measured with state probes over nine steps—equivalent to one inference. The total is 1.8 ms.

Figure 10 shows measured latency values while running on the Loihi 1 chip. The mean time for an inference step was 1.8 ms for Loihi 1 and 1.5 ms for Loihi 2(for information). As an additional result, the mean energy for inference was measured on the Loihi 2 chip, resulting in a dynamic energy consumption of 53 μJ.

### 3.3. Domain randomization

For domain randomization, the parameters of the friction cone model with μ and μ_2_ were randomized. *D*_1_ denotes a low friction environment with μ = μ_2_ = 0.2, while *D*_2_ denotes a high friction environment with μ = μ_2_ = 1. *D*_*R*_ represents a test environment with a random probability of sampling from the given set of environments. Table 1 shows the cross-evaluation of policies trained specifically for each environment (Π_1_ & Π_2_) and the policy trained with randomized environments (Π_*R*_) performing in different domains, where the values portrayed are the mean total sum of rewards of an episode and the performance profile, respectively.

**Table 1:** Domain randomization evaluation.

**Policy**	***D*_1_**	***D*_2_**	***D*_*R*_**
Π_1_	3.0 ± 3.6	1.6 ± 3.0	2.3 ± 3.4
Π_2_	2.3 ± 3.4	3.0 ± 3.7	2.7 ± 3.5
Π_*R*_	2.9 ± 3.6	2.8 ± 4.2	2.9 ± 3.9

All the values refer to the expected return and are in the same order of magnitude (1e + 5).

### 3.4. Learning complex tasks—injecting prior knowledge

When injecting prior knowledge with expert-guided learning, the time needed to reach a policy that consistently inserts the peg can be sped up by a factor of nearly five.

**Algorithm 1:**
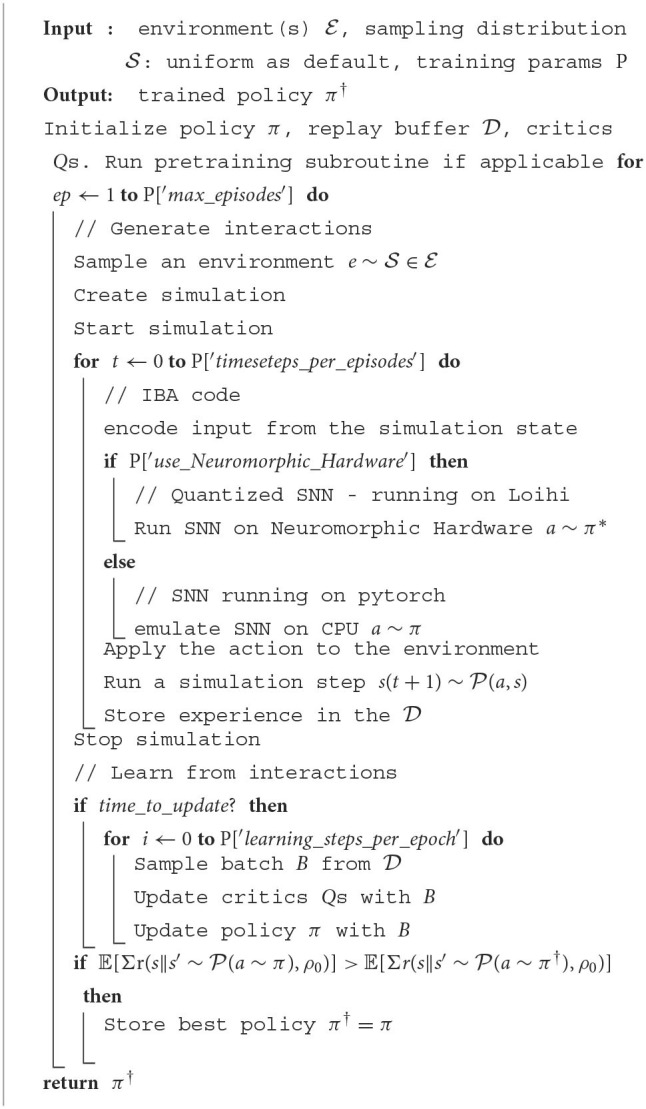
General learning approach-(Main Virtual Coach Script).

### 3.5. Consideration of neuromorphic hardware constraints

After training the model from Section 3.1, the performance measure was the insertion success rate. This value did not change after porting the SNN to neuromorphic hardware, and further considerations (such as those presented in Section 2.4.7) were not needed.

## 4. Discussion

We have presented a framework for training robust neurorobots in domains with variable parameters, as well as for carrying out inference using neuromorphic hardware. This approach uses the NRP, with emphasis on features such as the Virtual Coach and the Integrated Behavioral Architecture. With this approach, a simulated robot was trained to insert a peg into a hole with a small clearance based on only force-torque feedback. The results show 100% insertion success rate after 3 s. We also used domain randomization for addressing problems related to learning in environments with parametric uncertainties. The technique proved to reduce the sim-to-real gap when porting from simulation to a real robot. We document the process of porting these results to a real robot counterpart in a pending separate study.

This study is limited to model-free actor-critic architectures. Other training algorithms might create the need for restructuring the code; however, the main idea of the approach would be the same. Currently, it is not possible to learn during the episode, but this could be solved with use of threads to optimize resources and training time. Similarly, there is currently no support for multiple backends running in parallel, which would be an interesting feature for efficient implementations of distributed learning approaches highly parallelizable algorithms such as PPO (Schulman et al., 2017), or metalearning approaches (Beck et al., 2023). Those features represent some open new avenues for future work.

The results on the generic training show how the algorithm reaches a stable behavior with 100% success rate. The lack of smoothness is due to both the stochasticity of the environment and policy but also to the non-linear reward landscape. Notice how the variance is small at the beginning since all the returns are small and therefore similar for untrained policies. Once the training progresses, some runs would reach high rewards after insertion, while others would not, which generates high variances between runs. Finally, the variance decreases as a 100% insertion rate is reached, and all the returns are similar as there target is always reached. The remaining variance is due to differences in insertion time and other variables.

The results of the inference in neuromorphic hardware and system profiling show that the inference (controlling the robot) can be performed with low latency, low energy consumption, and without meaningful performance loss, namely, accurate inferences below 2*ms* with a dynamic energy consumption around 50μ*J*. A more detailed overview about the energy and time profiling for this specific setup is documented in the pending study mentioned above. A real robot counterpart is there controlled with the tools presented here, and more emphasis is set on the hardware.

The results from the domain randomization procedure show a consistent inter-domain overall improvement. The policy trained with domain randomization performs similarly to the policies trained for specific environments in both domains, while the specific policies have a significant performance drop while performing other tasks.

The results when injecting and relabeling prior expert knowledge show significant speed up for reaching a convergent policy in complex environments. The approach does not require a simulator that can be restarted at arbitrary states and resembles other LfD strategies (Argall et al., 2009). However, it requires to have an expert agent, a mapping function, under certain circumstances, this approach is equivalent to transferring knowledge via value functions.

For the sake of the implemented robotic task, quantization-aware training was not needed. One explanation for this might be the fact that we lead the training to learn robust policies, and therefore, the behavior was not sensible to the small changes in weights due to quantization. This could however be an important aspect for other learning environments.

Altogether, the NRP is arguably the most advanced integration platform for connecting robot simulations with in-silico brains currently available, and using adaptations as the one presented in this study expands the horizon of possible implementable setups. In this manuscript, we presented how we extend features such as including neuromorphic hardware platforms (Intel's Loihi Chips), utilizing multi-simulation learning algorithms (SAC), and adding elements as domain randomization to speed up research and move forward the frontiers in neurorobotics.

## Data availability statement

The code of the NRP experiment, the training and the inference can be requested to the author's correspondence.

## Author contributions

AA and LA contributed to the conception and design of this study. LA conceptualized and implemented the simulation environment, the learning approach, the training porting and inferring on neuromorphic hardware and further processes to obtain and visualize results, and wrote the first draft of the manuscript. AA leads the research group and contributed in conceptualization and management of the project. Both authors contributed to the manuscript, with the revision, proofread, and approval of the submitted version.

## Footnotes

^1^We will use the abbreviation NRP to refer to NRP 3. Although the NRP 4 is the latest release, we used exclusively v3 in this work.

^2^Gazebo is a well-known open source robotic 3D simulator.

^3^SDK developed by Intel for the Loihi 1 chip.

^4^The initial pose was located above the insertion pose with some added gaussian noise in all three coordinates. The mean and covariance of the noise distribution were treated as hyper parameters and have a strong influence in learning speed.

^5^Encoding in the context of a SNN refers to various methods to represent non-event-based information as spiking data. Real numbers can be represented as spiking-rates, spiking-times, or spiking-populations. For full detail refer to Dupeyroux et al. (2021).

^6^Notice that this is only possible when a function to map the state spaces is available $F:S_{expert}\to S_{agent}$. This mapping could be an approximation, however, no convergence analysis has been done concerning this.
